# Dual Identification: Trajectories to English Proficiency for English Learners with Autism Spectrum Disorder

**DOI:** 10.1007/s10803-023-05994-9

**Published:** 2023-05-12

**Authors:** Fernanda A. Castellón, Alexandra Sturm, Connie Kasari

**Affiliations:** 1 University of California, 760 Westwood Plaza 68–268, Los Angeles, CA 90024 USA; 2 Loyola Marymount University, Los Angeles, USA

**Keywords:** Special education, Autism, English learners, Educational placement, English proficiency

## Abstract

**Background:**

There are an increasing number of English Learners (EL) served in schools, including children with Autism Spectrum Disorder (ASD). However, little is known about students who receive school-based services as EL and under autism eligibility.

**Purpose & Methods:**

The present study aimed to examine the sociodemographic characteristics, time to English Language Proficient status by survival analysis, and predictors of English fluency utilizing a logistic regression for dually identified EL and autism eligible students in a large urban school district during the 2011–2019 academic school years.

**Results:**

Overall, dually identified students (N = 849) educated in segregated settings (N = 372) became English proficient at lower rates and at older ages than students included in general education (N = 477).

**Conclusion:**

Students placed in segregated special education classrooms were significantly less likely to achieve English Language Proficient classification. The present study begins to illustrate the time to English proficiency of dually identified students and the potential impact it has on their educational opportunities.

## Introduction

In the past 30 years, the United States has received an influx of international immigrants from diverse countries, linguistic origins, and ethnic groups (Glick & Hohmann, 2007). As a result, there are a growing number of students who have at least one foreign-born parent and who speak a language other than English at home (Aud et al., 2010; Fry, 2007; Thompson, 2017). Students who are identified by their caregiver as having a home language other than English and who are not proficient in speaking, writing, and listening on an initial English language proficiency assessment are designated as English Learners (EL) in public educational settings (Boyle et al., 2010; CDE, 2021b, 2022). Students participating in the special education system are at risk for poorer English proficiency and becoming Long Term English Learners (CDE, 2022; Okhremtchouk et al., 2018, 2007; Slama et al., 2017; Thompson, 2017). One of the fastest growing populations of students qualifying for special education services are those students receiving special education services under the disability eligibility of autism (Maenner & Durkin, 2010; Newschaffer et al., 2005; Author & Author, 2021). In addition to services received to support English language proficiency, ELs who meet eligibility requirements for a documented disability (e.g., autism) may also qualify for special education services. Autism is defined as a neurodevelopmental disability that is present from an early age and characterized by difficulties in communication, behavior, and restricted interests/repetitive behaviors (APA, 2013). In 2018–2019, approximately 8% of EL students were dually identified as eligible for special education services under the disability criteria of autism (U.S. Department of ED, 2019). However, there is no current data on the sociodemographic characteristics of dually identified students, their time to English proficiency, and predictors of proficiency.

A significant barrier to pinpointing the educational needs of EL autistic students is the difficulty in parsing the difference between disability-associated language difficulties and the need for English language-specific development. There are various risk factors associated with remaining as an EL student such as late identification of both EL status or disability, and lack of appropriate ELD instruction, special education services, or accessibility to reclassification assessments. The literature in this area has found a paradoxical representation of EL students with disability eligibilities such as intellectual disability (ID) and speech learning delay (SLD), claiming that such students are either wrongly over- or underrepresented (Artiles et al., 2005; De Valenzuela et al., 2006; Hibel & Jasper 2012; Rueda & Windmueller, 2006; Sullivan, 2011). Hibel and Jasper (2012) illustrated that students from immigrant families were significantly more likely to be labeled as EL before they were screened for a disability. Work from Sullivan (2011) is consistent with Hibel and Jasper (2012) as they identified a similar trend that showed that when compared to their White peers, EL students were increasingly identified as disabled and transferred to a special education placement as they progressed through the school system.

Services for autism eligibility and for English language instruction can both involve inclusion in the general education curriculum, or instruction in segregated settings. For EL students, teachers receive guidance and tools to target English Language Development (ELD) standards through *integrated instruction*, in which ELD standards and state-adopted academic standards are targeted simultaneously (CDE, 2021a), and *designated instruction*, in which ELD standards are targeted during a protected time in the regular school day (CDE, 2021). When students receive designated ELD instruction, they may be required to miss general education instructional time to receive ELD instruction in a separate setting. Students eligible to receive services under autism may also be educated in inclusive settings (i.e., general education settings) or segregated settings (e.g., special day class/ special education school) or a unique combination of the two. Currently, 40% of students receiving services under autism eligibility are educated in inclusive settings, leaving more than 60% of students in segregated settings (Morningstar et al., 2017). Students who are educated in segregated school settings are more likely to have limited access to the general education curriculum, or modifications to the general education curriculum, which can further hinder academic progress (Gee et al., 2020).

The purpose of segregated special education classrooms is to deliver individualized and adapted instruction by special educators, therapists, and other special education resources to those students with disabilities who would not successfully learn in general education classrooms (Fisher & Meyer, 2002). These educational placements are determined by the educational team and are meant to be aligned with IDEA’s Least Restrictive Environment (LRE) requirement, meaning that students with disabilities should be educated in general education classrooms as much as possible (IDEA, 2005). Research has shown that this is not the case for students with significant disabilities, from racial and ethnic minority backgrounds, and ELs as they have historically been placed in segregated special education settings (Ferri & Connor, 2002). There have long been reports of over-representation of EL students in special education classrooms with the evidence hinging on early EL services that ultimately result in later special education designation and the delay of necessary intervention services (Artiles et al., 2005; Sullivan 2011). However, most data only include students with intellectual disability (ID), speech language delay (SLD), and learning disability (LD) from across the United States, with no current literature documenting the language proficiency outcomes of autistic EL students (Artiles et al., 2005; Hibel & Jasper 2012; Rueda & Windmueller, 2006; Sullivan, 2011).

Although some may argue the need for the concentration of services in a segregated setting for students with disabilities (Baker & Zigmond, 1990; Fuchs & Fuchs, 1994) and EL students (CDE, 2021a), the segregation of students based on linguistic capabilities or disability can negatively impact students. Linguistic segregation and teacher implicit bias can negatively impact student access to rigorous grade level material (CDE, 2019; Gandara et al., 2008). Such experiences can reduce the academic opportunities of EL students that are consequential for strong oral skills, literacy competence, academic achievement, personal expectations, school engagement, and ultimately impact long-term career-related success (Clark-Gareca et al., 2020; Slama, 2012; Scarcella, 2003; Snow & Kim, 2007; Umansky, 2018). Students with disabilities who remain in segregated special education settings are less likely to receive a high school degree, pursue higher education, and have low rates of employment (CDE, 2022). In contrast, research has demonstrated successful participation, learning, friendship development, and achievement of IEP objectives in general education settings (Fisher et al., 2002; Hunt et al., 1994; Freeman & Kasari, 1998; Kasari et al., 2012; Webster & Carter 2007). For students who are both autistic and ELs, being in a segregated special education setting while also needing both special education services and English development services may result in an augmented negative impact as some areas of need (i.e., language development, social interactions, adaptive skills, academic standards) may be prioritized over others and therefore not targeted to their fullest extent as they would be in an inclusive general education setting.

Not only is the transition to English proficiency important for student academic outcomes, the *timing* of reclassification to English proficient is also highly predictive of a student’s academic opportunities. Students who become English proficient by middle school perform academically similarly to native English speakers (Thompson, 2017) and better than students who remain EL. In contrast, those who remain as EL after the elementary grades are at an increased risk of dropping out of high school and failing to pursue higher education (Sheng, Christine, & Anderson, 2011; Kieffer et al., 2008). Those most at risk for remaining EL are those who are native Spanish speakers, students whose parents have limited formal education levels, and those who qualify for Free and Reduced-Price Lunch (FRL) (Thompson, 2017; Slama, 2014). These students become English proficient at older ages or never reach English proficiency while in school (Umansky & Reardon, 2014) and are categorized as Long-Term English Learners (CDE, 2022).

## Current Study

Prior literature documents barriers EL students with disabilities face in accessing critical educational supports and services and achieving English Language Proficient classification. As both EL and autistic students are individually the fastest growing populations of students served in public schools, it is imperative to begin to analyze the trajectories of English proficiency and predictors associated with retaining EL status among autistic students (Umansky, 2018; Stichter et al., 2017). The present study aims to:

1. Characterize elementary-aged English learner students served under autism eligibility in a large urban school district.
2. Determine the mean age at which autistic students classified as EL become English language proficient.
3. Analyze potential predictors of EL proficiency for autistic EL students at their time of reclassification to English Proficient.

## Methods

### Participants

The present study performed secondary data analysis of student special education individualized education program (IEP) administrative records from a large urban school district in Southern California. A student was included in the present study if the student (1) had a primary eligibility of autism, (2) was classified as an English Learner (EL) during at least one of the available observation years, (3) was enrolled in TK-5th grade at their first observation in the 2011–2012 academic school year, and (4) had at least one annual observation during the available academic years (2011–2012 to 2018–2019). Of the N = 29,356 autistic students who met the study inclusion criteria, N = 7532 students were also classified as EL during at least one of their annual observations (Aim 1). To address Aims 2 and 3, the sample was further reduced to include students who had at least two observations (N = 2014 students, N = 13,110 observations; mean observations per student = 6.51). The study was determined exempt from IRB review as data were de-identified and the study was not preregistered with an analysis plan on an independent, institutional registry.

### Measures

Student IEP records included basic demographic information for each student including age, grade level, household income, free and reduced lunch (FRL) status (FRL or no FRL), in addition to eligibility (e.g., autism) school type (public day school, non-public day school, special education center or facility, and charter school), and district region (North, East, West, South) and the associated median neighborhood income (<$40,000, [$40,000, $79,999], [$80,000, $119,999], and ≥$120,000) (United States Census Bureau, 2016). In addition, an inclusion variable was calculated based off district records reporting the percentage of time spent in either GE (general education classroom- GE) or SE (special education classroom- SE), where 50% or more of the school day spent in GE was categorized as an inclusive placement.

### Analyses

#### Sample Baseline Characteristics of Dually Identified Students

To quantify the sociodemographic characteristics of all EL and ELP served under an eligibility of autism across academic school years, a sample of students who had at least one observation (N = 7532) was utilized. Univariate statistics were calculated by ethnicity, educational placement, median household income, and free and reduced lunch status.

#### Age of EL Proficiency

To determine the age at which students were most likely to transition to English Language Proficient (ELP), a sample of students who had at least two observations across all school years was utilized (N = 2014). Kaplan-Meier survival analysis (David & Mitchel, 2012; Therneau, T. M. & Grambsch, P. M., 2000) were conducted using survival packages in R (R Core Team, 2021; KMsurv, survival, Therneau, 2021). Survival analysis investigates the expected length of time until an event of interest occurs (i.e., losing EL identification) or does not occur (Singer & Willett, 1991). Eligible students were observed until the end of the study when they either acquired English proficiency or are labeled as “censored” if they remain classified as EL. The survival times to English proficiency for autistic EL students were illustrated through Kaplan Meier Curves (N = 2014) showing the length of time EL identification survived. The survival time of EL identification was calculated utilizing the age variable and the dichotomous variable of English proficiency which indicated if a student was identified as EL or if they had reached ELP during the available observation years. For those participants who became ELP, the age when they lost their EL classification was recorded.

#### Predictors of EL Proficiency

Finally, to identify potential student-level predictors associated with the timing of ELP classification, multiple linear regression was utilized for a sample of students who became ELP at any point throughout the available academic school years (N = 849). Previous work has identified relationships between educational placement, ethnicity, and income levels among students residing in urban settings (Brock & Schaefer, 2015), therefore the current study included similar predictors while also looking to text novel predictors. Predictors included educational placement the year before reaching ELP, ethnicity, gender (male or female), median income category, and FRL status. Outcome was the age when a student reached ELP.

## Results

### Sample baseline characteristics of dually identified students

Across all available observation years (2011–2019), N = 7532 students were served under an eligibility of autism and were also classified as EL at least one time across the observation years. The average age of first observation was 5.73 years (SD = 2.11). N = 1902 students had a general educational placement (25%), and a large majority were Hispanic/Latinx students (87.3%). N = 4576 (87.5%) students qualified for Free or Reduced Lunch (FRL), and N = 3282 (43.5%) students had a median annual income of less than $40,000 (USD) aligning with U.S. Census Federal Poverty Line (FPL) guidelines of 138% of the poverty threshold, qualifying as low-income (United States Census Bureau, 2019). At the first observation, N = 5251 (78.7%) students were already identified as EL, while N = 1607 (21%) students were unidentified as EL at their first observation but were identified as EL at a later observation. Chi-square tests between the two groups showed significant differences among the categories of mean age, multiple race/ethnic categories (Hispanic, Asian, White, and African American; *p* < 0.0001), FRL (*p* < 0.0001), grade levels (*p* < 0.0001), educational placement (*p* < 0.001), inclusive placement (*p* = 0.0019), academic curriculum (*p* < 0.0001) and the median income categories of $40,000-$80,000 (*p* = 0.001) and $80,000-$120,000 (*p* = 0.004) (see Table 1 for demographic information).

**Table 1:** Sociodemographic Characteristics at Baseline

	Total *(N = 7532)*	EL (*n* = 5925)	Unidentified EL (*n* = 1607)	*P value*	
Mean Age (SD)	5.73 (2.11)	4.05 (1.23)	6.18 (2.07)	< 0.05	
Race/ Ethnicity					
Hispanic	6577 (87.3%)	5251(88.6%)	1326 (82.5%)	< 0.0001	
Asian	499 (6.63%)	361 (6.10%)	138 (8.59%)	0.0003	
White	324 (4.30%)	226 (3.81%)	98 (6.10%)	< 0.0001	
Filipino	81 (1.08%)	57 (0.96%)	24 (1.50%)	0.067	
African American	27 (0.36%)	14 (0.24%)	13 (0.81%)	0.0007	
Native American	7 (0.09%)	6 (0.10%)	1 (0.06%)	1	
Pacific Islander	3 (0.04%)	1 (0.02%)	2 (0.12%)	0.117	
Mixed/Multi	4 (0.05%)	3 (0.05%)	1 (0.06%)	1	
Unknown	10 (0.13%)	6 (0.10%)	4 (0.25%)	0.236	
Gender					
Female	1145 (15.2%)	871(14.7%)	274 (17.1%)		
Male	5856 (77.7%)	4541(76.6%)	1315 (81.8%)	0.276	
FRL					
Yes	4576 (60.8%)	3856 (65.1%)	720 (44.8%)		
No	2956 (39.2%)	2069 (34.9%)	887 (55.2%)	< 0.0001	
Grade					
Kindergarten/ Transitional Kinder	1593 (21.2%)	1396 (23.6%)	197 (12.3%)	< 0.0001	
Preschool	2555 (33.9%)	1318 (22.2%)	1237 (77.0%)	< 0.0001	
1st	1233 (16.4%)	1133 (19.1%)	100 (6.22%)	< 0.0001	
2nd	713 (9.47%)	668 (11.3%)	45 (2.80%)	< 0.0001	
3rd	562 (7.46%)	544 (9.18%)	18 (1.12%)	< 0.0001	
4th	484 (6.43%)	474 (8.00%	10 (0.62%)	< 0.0001	
5th	392 (5.20%)	392 (6.62%)	0	< 0.0001	
Educational Placement					
GE	1902 (25.3%)	1671(29.9%)	231(14.4%)		
SE	5242 (69.6%)	3915 (70.1%)	1327 (82.6%)	< 0.001	
Inclusive Placement					
Inclusion	3080 (40.9%)	2435 (41.1%)	645 (40.1%)		
Non-Inclusion	3921 (52.1%)	2977 (50.2%)	944 (58.7%)	0.0019	
Academic Curriculum					
Core Curriculum	6518 (86.5%)	5009 (84.5%)	1509 (93.9%)		
Alternative Curriculum	862 (11.4%)	778 (13.1%)	84 (5.23%)	< 0.0001	
Median Income Categories					
Less than $40,000	3282 (43.6%)	2559 (43.2%)	723 (45.0%)	0.196	
$40,000-$80,000	3455 (45.9%)	2661(45.0%)	0	0.001	
$80,000-$120,000	184 (2.44%)	129 (2.18%)	55 (3.42%)	0.004	
$120,000	1 (0.01%)	1 (0.02%)	0	1	

*Note.* GE = general education, SE = special education classroom, FRL = free or reduced-priced lunch

### Age of EL proficiency

**Fig. 1:**
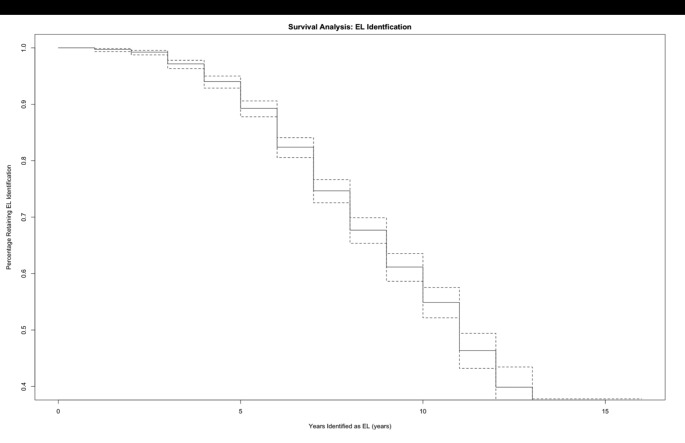
Product-Limit Survival Estimates for Autistic EL Students. (*Note*. Kaplan-Meier survival curve (solid line) and 95% confidence intervals (dashed lines) for years of EL identification (English Learner) among students who had at least two observations (N = 2014))

Number of available observations ranged from 1 to 8 (M = 6.5) and N = 1018 students (51%) had observations for all eight years. Figure 1 illustrates “survival time” for EL classification, where the x-axis represents the number of years a student was classified as an EL, and the y-axis represents the likelihood that a student’s EL classification would remain. Only 38.4% (N = 774) of students initially classified as EL had achieved English proficiency by the time of their last observation. The mean age at which dually identified students transitioned to English proficient was approximately 12.3 years (SD = 2.47) with an average of 7.14 (SD = 2.49) years as an EL. Table 2 illustrates the survival of EL identification based on Kaplan-Meier estimates. The Kaplan Meier curve (Fig. 1) indicated that the probability of classification as EL decreased as students aged.

**Table 2:** Multiple Linear Regression

Predictors	Estimates	SE	95% CI		*p*
			*LL*	*UL*	
Fixed Effects					
Intercept	8.67	1.87	4.97	12.4	**< 0.001**
Placement before ELP	0.82	0.31	0.21	1.43	**0.009**
Ethnicity [Asian]	1.19	2.06	-2.89	5.27	0.564
Ethnicity [Filipino]	2.27	2.24	-2.16	6.70	0.313
Ethnicity [Hispanic]	1.76	1.98	-2.15	5.68	0.375
Ethnicity [White]	0.84	2.15	-3.42	5.10	0.696
Income Category (Less than $40,000)	-0.12	0.326	-0.76	0.53	0.717
Income Category ($80,000-$120,000)	-0.37	0.66	-1.67	0.93	0.577
FRL	-0.59	0.53	-1.63	0.45	0.264
Gender	0.62	0.50	-0.36	1.60	0.216

*Note.* ELP = English language proficient, FRL = free or reduced-priced lunch

### Predictors of EL proficiency

Multiple linear regression analyses demonstrated that type of educational placement (GE vs. SE) before the student became ELP was a significant predictor of the age when a student lost their EL identification and therefore became ELP (β = 8.67, SE = 1.87, *p* = 7.98e-06). Placement in a special education classroom was associated with significantly later age at English proficiency (β = 0.823, SE = 0.31, *p* = 0.009). Ethnicity (Asian β = 1.19, SE = 2.06, *p* = 0.56, Filipino β = 2.27, SE = 2.24, *p* = 0.31, Hispanic β = 1.76, SE = 1.98, *p* = 0.37, and White β = 0.84, SE = 2.15, *p* = 0.69), household income (<$40,000 β = -0.12, SE = 0.33, *p* = 0.72 and [$80,000,- $120,000 β = -0.37, SE = 0.66, *p* = 0.58 ), receiving FRL (β = -0.65, SE=, *p* = 0.218) and gender (β = 0.62, SE = 0.496, *p* = 0.22) did not predict age when a student became ELP (see Table 3 for all regression parameters).

**Table 3:** Survival of EL identification based on Kaplan-Meier estimates

EL identification time (yr)	No. of students at risk	No. events	Survival Rate	Std. Error	Lower 95% CI	Upper 95% CI
1	1993	6	0.997	0.00123	0.993	0.999
2	1966	9	0.992	0.00195	0.987	0.995
3	1906	40	0.972	0.00378	0.963	0.978
4	1829	59	0.940	0.00543	0.929	0.950
5	1719	87	0.893	0.00716	0.878	0.906
6	1533	118	0.824	0.00898	0.806	0.841
7	1343	126	0.747	0.01045	0.725	0.766
8	1092	102	0.677	0.01153	0.654	0.699
9	818	79	0.612	0.01255	0.586	0.636
10	557	57	0.549	0.01373	0.522	0.575
11	340	53	0.463	0.01584	0.432	0.494
12	172	24	0.399	0.01832	0.363	0.434
13	49	9	0.325	0.02665	0.274	0.378

*Notes.* Number of students at risk represents the number of autistic students who were identified as English Learners; the number of events gives the number of students who became English Language Proficient

## Discussion

The current study utilized administrative data from a large urban school district to evaluate characteristics of students with dual eligibility (autism and EL), and the timing and predictors of English proficiency among autistic students initially classified as English Learners. The strongest predictor of becoming ELP for EL autistic students was their educational placement in the year before they achieved English proficiency.

### Age of Reaching ELP

EL autistic students in the present study who did reach ELP (38.4%) were, on average, 12.3 years of age (SD = 2.47), aligning roughly with 7th grade. California Statewide reports show that on average, 88% of the EL student population becomes English proficient by 8th grade (Slama et al., 2017). However, in the present sample less than half (41.9%, N = 774) of the students who achieved English proficient classification within the observation period did so by 8th grade (Slama et al., 2017). The majority of the students who did not reach ELP were Latinx (91.1%), male (83.7%), and from low socioeconomic status (86.1%). Reclassification as English proficient by the end of middle school is of great significance due to the educational opportunities that are available in high school. Due to the need for ELD support, EL students continue to receive ELD instruction as appropriate in both integrated and designated instructional settings (CDE, 2021a). As a result, EL students are often not eligible to enroll in specialized courses such as electives, honors, or even Advanced Placement courses due to their EL status and need of ELD support (Callahan & Shifrer, 2016; Zuniga et al., 2005).

Promoting equitable practices that will allow students to reach English proficiency is a priority among educators as the rates of EL students continues to grow. EL students experience high rates of high school dropout due to their low academic achievement, putting them at risk for poorer employment outcomes and poverty (Sheng et al., 2011; Kieffer, 2008). The risks for autistic EL students are even greater as recent reports show that as autistic students leave high school, they have minimal access to supportive services, low career-readiness skills, and a minority pursue post-secondary education (Shattuck et al., 2012a, b; Howlin & Moss, 2012; Friedman et al., 2013). Students who undergo extensive testing to be identified as both autistic and EL have educational needs that are distinct from those of non-EL autistic students and non-autistic EL students. Finding the balance between addressing autism-specific educational needs such as language production and EL-specific needs such as language comprehension may be challenging for educators. Overall, the low rates of autistic EL students who become ELP relative to published statewide estimates for EL students underscores the additional complexity of dual identification. In the past, EL identification and autism eligibility have been explored separately. However, the current data suggest that the intersectionality of language proficiency and autism should be examined concurrently.

### Predictors of ELP

The strongest predictor of becoming ELP was a student’s educational placement in the year before they reached English proficiency. Students educated segregated (SE) classes reached English proficiency at older ages than those educated in GE. The explanation of the relationship between placement and English proficiency cannot be answered using school administrative data. Indeed, it is unclear if student-level characteristics (e.g., autism symptom severity, expressive language ability in heritage language) that may have informed a segregated placement may best explain delayed English language acquisition. Alternatively, the inaccessibility of the ELPAC to students with disabilities may also explain delayed proficiency (Abedi, 2006; Bailey & Carroll, 2015). A new Alternate ELPAC has been developed and will be implemented in schools this academic school year that better accommodates students with disabilities (CDE, 2022). Finally, because inclusive settings are associated with better academic and social outcomes compared to segregated settings (Rujis & Peetsma, 2009; Hunt & Goetz, 1997), another potential explanation is that general education settings could promote the English development of autistic EL students by providing more opportunities for English language practice. Inclusive settings have been shown to not only promote academic skills such as language and functional communication, but also social skills such as social interaction, play, and socio-emotional skills (Wehmeyer et al., 2021; Cioè-Peña, 2017; Freeman & Kasari, 1998). These are all opportunities where EL students are able to practice and develop their English proficiency through interacting with English-proficient peers (Artiles et al., 2005). As inclusive placements increasingly become an educational standard for students with disabilities, dually identified EL students, irrespective of student-level characteristics, will benefit from more contact with English-proficient peers.

Although inclusive settings are viewed as advantageous by experts, racial and linguistic minority autistic students have historically been placed in non-inclusive special education settings at higher rates than students from White middle-class backgrounds (Ferri & Connor, 2005). Ethnicity was not a significant predictor of English proficiency for this specific study, where more than 80% belonged to an ethnic minority group. However, both teachers and scholars need to remain cognizant of the well-documented connection between ethnicity, social-economic status, immigrant family background, urban settings, and the lack of access to inclusive educational placements (Brock & Schaefer, 2015; Cioè-Peña, 2017; Kim et al., 2014; Rueda et al., 2002). Both EL students and autistic students are placed in specific learning tracks that individually influence the future of the student. This iterative cycle of barriers to general education, current grade level instruction, and social interaction with peers have long-term educational and life outcomes that affect autistic individuals and their families (Stahmer & Ingersoll, 2004). Due to the barriers that are present for autistic EL students, becoming English proficient is a vital skill that can directly influence their futures. Therefore, for autistic EL students, becoming English proficient should be a priority to be better prepared for a successful transition into adulthood.

## Limitations

The current study had various limitations which future research should address. First, the current data did not include English Learners with no disability identification, therefore no comparison between autistic and non-autistic groups could be made. Secondly, we did not have access to student IEP goals or academic records and therefore we could not assess the extent to which certain student characteristics (e.g., presence of co-occurring disabilities, IQ, special education supports) may have contributed to variability to timing of ELP. We anticipate that students with extensive communication difficulties (e.g., those who rarely use any spoken language to communicate) may be less likely to be classified as English proficient and less likely to be placed in general education classrooms. This also raises concern around how language ability and English proficiency is evaluated among autistic EL students with complex communication needs, particularly given that nearly 30% of autistic children are considered “minimally verbal” at age 5 years (Tager-Flusberg & Kasari, 2013). Finally, the current study followed a cohort-like type of analysis for one large-urban school district and therefore generalizations should be made with caution to populations across other districts.

## Future Directions

Future research should continue to examine additional predictors of interest among EL autistic students to English proficiency such as autism symptom severity and child’s expressive and receptive language in both English and their heritage language. There is growing literature showing that one in four autistic children are bilingual (Trelles & Castro, 2019) therefore EL autistic students may be receiving autism-language services focused on language production, when in fact they should be focused on language comprehension. The opposite may also be true, but by teachers having additional information about a child’s language level in their heritage language, they may be better able to pinpoint a child’s specific language needs. In addition, due to the hypothesis that EL autistic students may have more opportunities to practice their English in a general education classroom, future research should look to examine the language learning opportunities that are available in each classroom setting, e.g., through behavioral coding of teacher and student interactions.

## Conclusion

In summary, the growing number of autistic English Learners calls for further longitudinal research to document the rates at which such students reach English proficiency. This process may be further complicated by the intersectional identities of EL learners as members of ethnic racial minorities, linguistic minorities, and the disabled community. By documenting the prevalence of autistic EL students, further research may begin to analyze the facilitators and barriers that make up the English proficiency process for students on the autism spectrum.
